# Supplementary material for: Multimodal evidence for a mechanistic model of working memory deficits in schizophrenia
Source: medRxiv. 2026 Jun 30:2026.06.24.26356367. Preprint. [Version 1] doi: 10.64898/2026.06.24.26356367 (PMC13345478; doi:10.64898/2026.06.24.26356367)
Supplement: Supplementary file 1 [file NIHPP2026.06.24.26356367V1-supplement-1.pdf]

medRxiv preprint doi: https://doi.org/10.64898/2026.06.24.26356367; this version posted June 30, 2026. The copyright holder for this preprint (which was not certified by peer review) is the author/funder, who has granted medRxiv a license to display the preprint in perpetuity.
It is made available under a CC-BY 4.0 International license .

Supplementary figures and tables

medRxiv preprint doi: https://doi.org/10.64898/2026.06.24.26356367; this version posted June 30, 2026. The copyright holder for this preprint (which was not certified by peer review) is the author/funder, who has granted medRxiv a license to display the preprint in perpetuity.
It is made available under a CC-BY 4.0 International license .

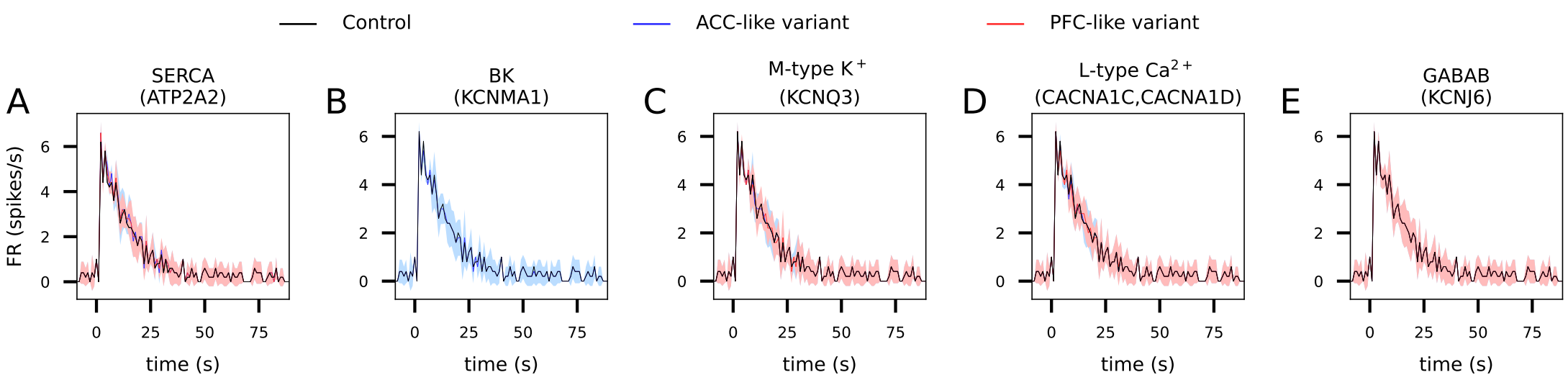

Figure S1: **Expression-level changes of ATP2A2, KCNMA1, KCNQ3, CACNA1C, CACNA1D, and KCNJ6 associated with SCZ had little effect on the response to a WM-inducing stimulus**. **A**–**E**: The single-neuron FR response to a WM-inducing stimulus (see Fig. 2A–E) was simulated under alterations of a single SCZ-associated ion channel as suggested by post-mortem expression data from PFC (red) and/or ACC (blue). The rate of the SERCA pump removing the Ca^{2+} from the cytosol (A), the conductance of large-conductance Ca^{2+}-activated BK channel (B), M-type K^+ channel (C), L-type Ca^{2+} channel (D), or the $GABA_B$R-activated GIRK channel (E) was altered according to the expression data of the underlying SCZ-associated genes.

medRxiv preprint doi: https://doi.org/10.64898/2026.06.24.26356367; this version posted June 30, 2026. The copyright holder for this preprint (which was not certified by peer review) is the author/funder, who has granted medRxiv a license to display the preprint in perpetuity.
It is made available under a CC-BY 4.0 International license .

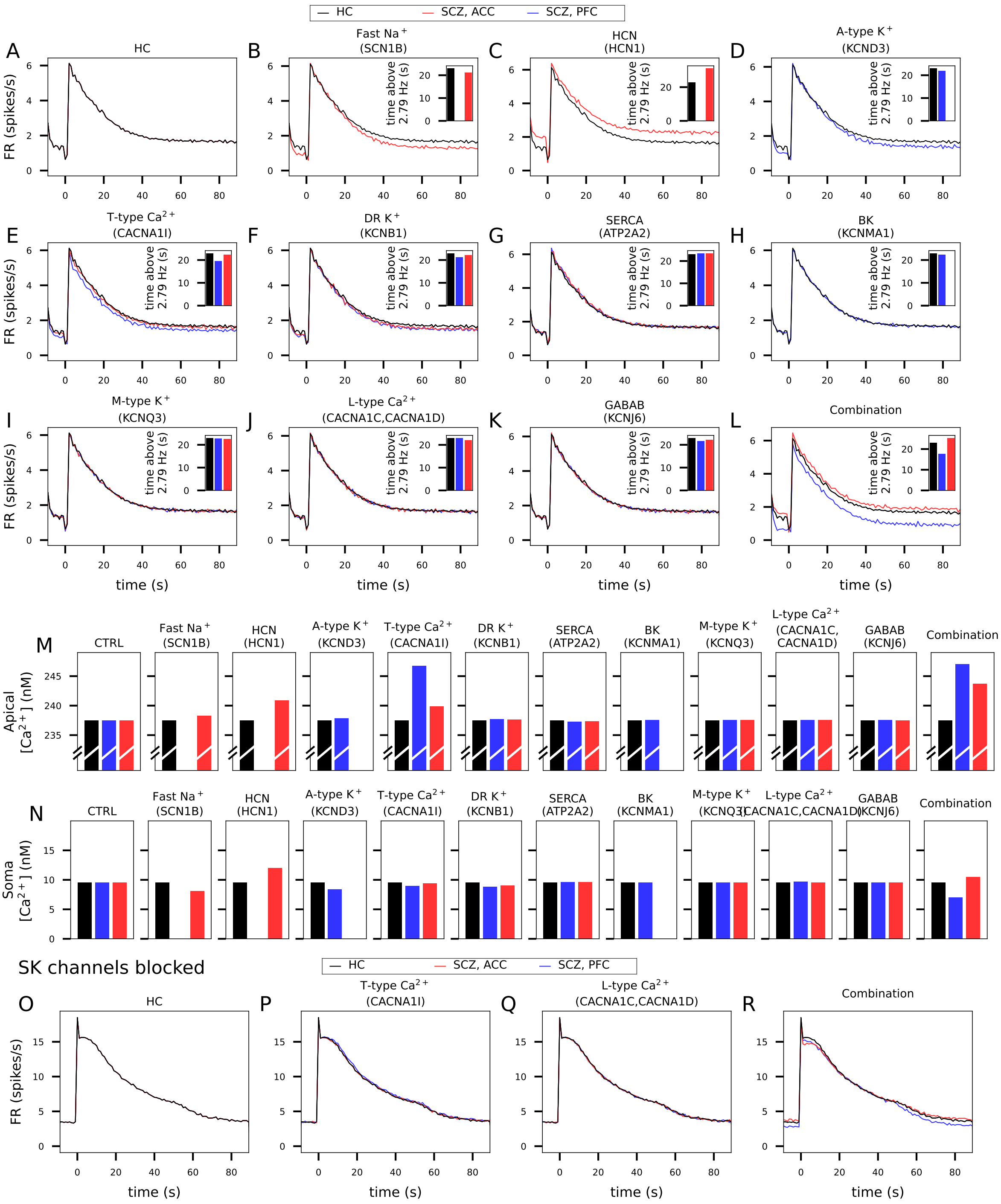

Figure S2: **Altered expression of SCN1B, HCN1 and KCND3 had large effects on sustained network activity and that of CACNA1I had a moderate effect on the network response to the WM-inducing stimulus, while other variants had little effect, and the inhibitory effects of CACNA1I overexpression were mediated by SK currents.** **A**: The firing rate of pyramidal cells in the default model. **B–K**: The effects of the population-averaged alterations (as measured in the ACC (blue) or PFC (red)) of expression of SCN1B (B), HCN1 (C), KCND3 (D), KCNB1 (E), CACNA1I (F), ATP2A2 (G), KCNMA1 (H), KCNQ3 (I), CACNA1C+CACNA1D (J), and KCNJ6 (K) on the firing activity caused by the WM-inducing stimulus. **L**: The effects of combined variants of Fig. 2K replotted for reference. **M–N**: The baseline Ca^{2+} concentrations (measured 0–3 s before the WM-inducing stimulus) in the furthermost apical dendrite (M) and in the soma (N) in the simulations of panels (A)–(L). **O**: The firing rate of pyramidal cells in the model where SK channels were blocked but no other ion-channel alterations were present. **P–Q**: The firing rate of pyramidal cells in the model where SK channels were blocked and the T-type (P) or L-type (Q) Ca^{2+} channel conductances were upscaled according to CACNA1I (P) or CACNA1C and CACNA1D (Q) expression data in SCZ. **R**: The firing rate of pyramidal cells in the model where SK channels were blocked and the combinations of ion-channel expression alterations were present.

medRxiv preprint doi: https://doi.org/10.64898/2026.06.24.26356367; this version posted June 30, 2026. The copyright holder for this preprint (which was not certified by peer review) is the author/funder, who has granted medRxiv a license to display the preprint in perpetuity.
It is made available under a CC-BY 4.0 International license .

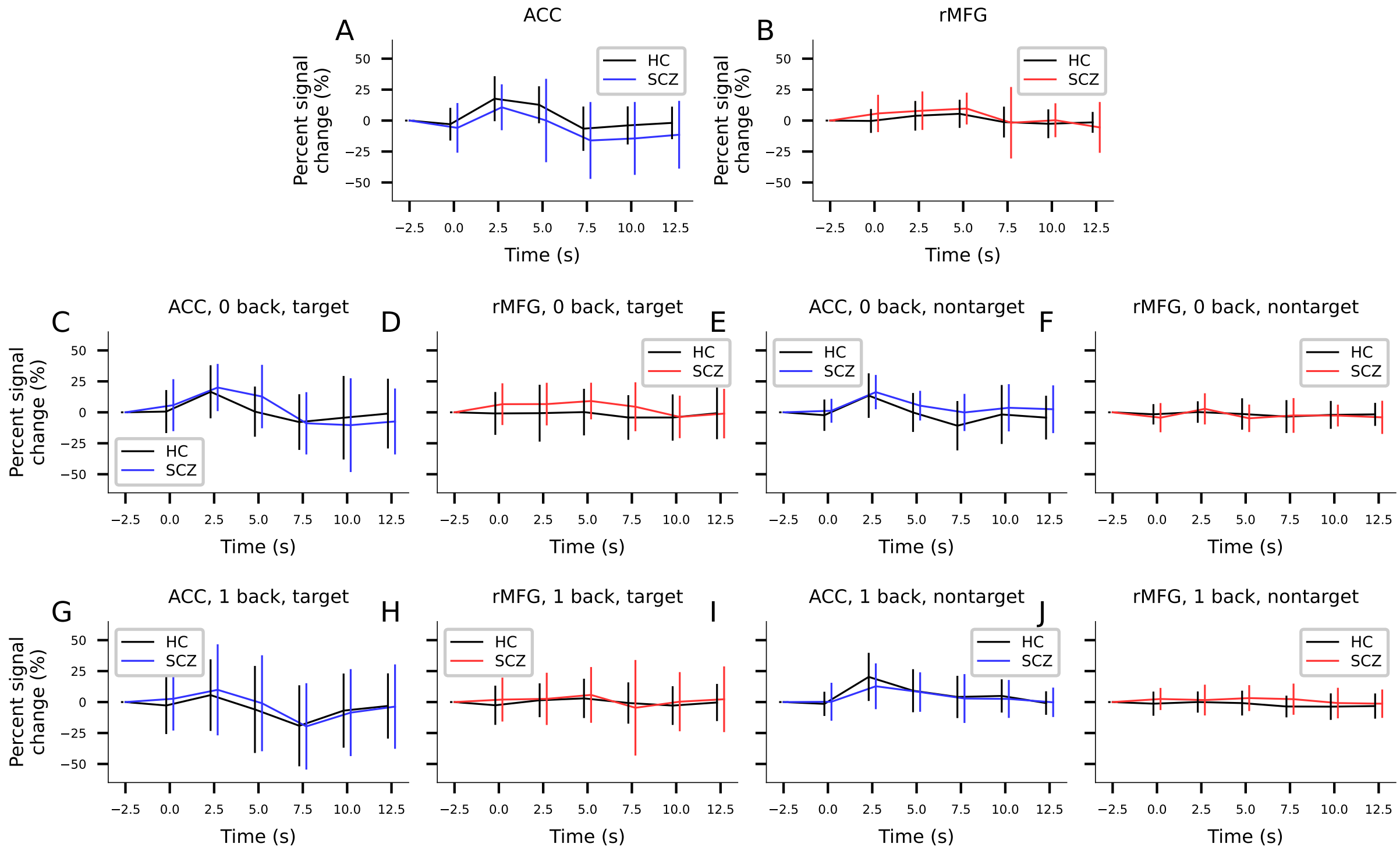

Figure S3: **The hemodynamic responses to nontargets in the 2-back task and the responses to targets or nontargets are not significantly different between participants with SCZ and HCs**. **A**–**B**: Hemodynamic responses in ACC (A) and PFC (B) in response to *non*target stimuli in the 2-back task. **C**–**F**: Hemodynamic responses in ACC (C,E) and PFC (D,F) in response to target (C–D) or nontarget (E–F) stimuli in the 0-back task. **G**–**J**: Hemodynamic responses in ACC (G,I) and PFC (H,J) in response to target (G–H) or nontarget (I–J) stimuli in the 1-back task. See Fig. 3J–K for details.

medRxiv preprint doi: https://doi.org/10.64898/2026.06.24.26356367; this version posted June 30, 2026. The copyright holder for this preprint (which was not certified by peer review) is the author/funder, who has granted medRxiv a license to display the preprint in perpetuity.
It is made available under a CC-BY 4.0 International license .

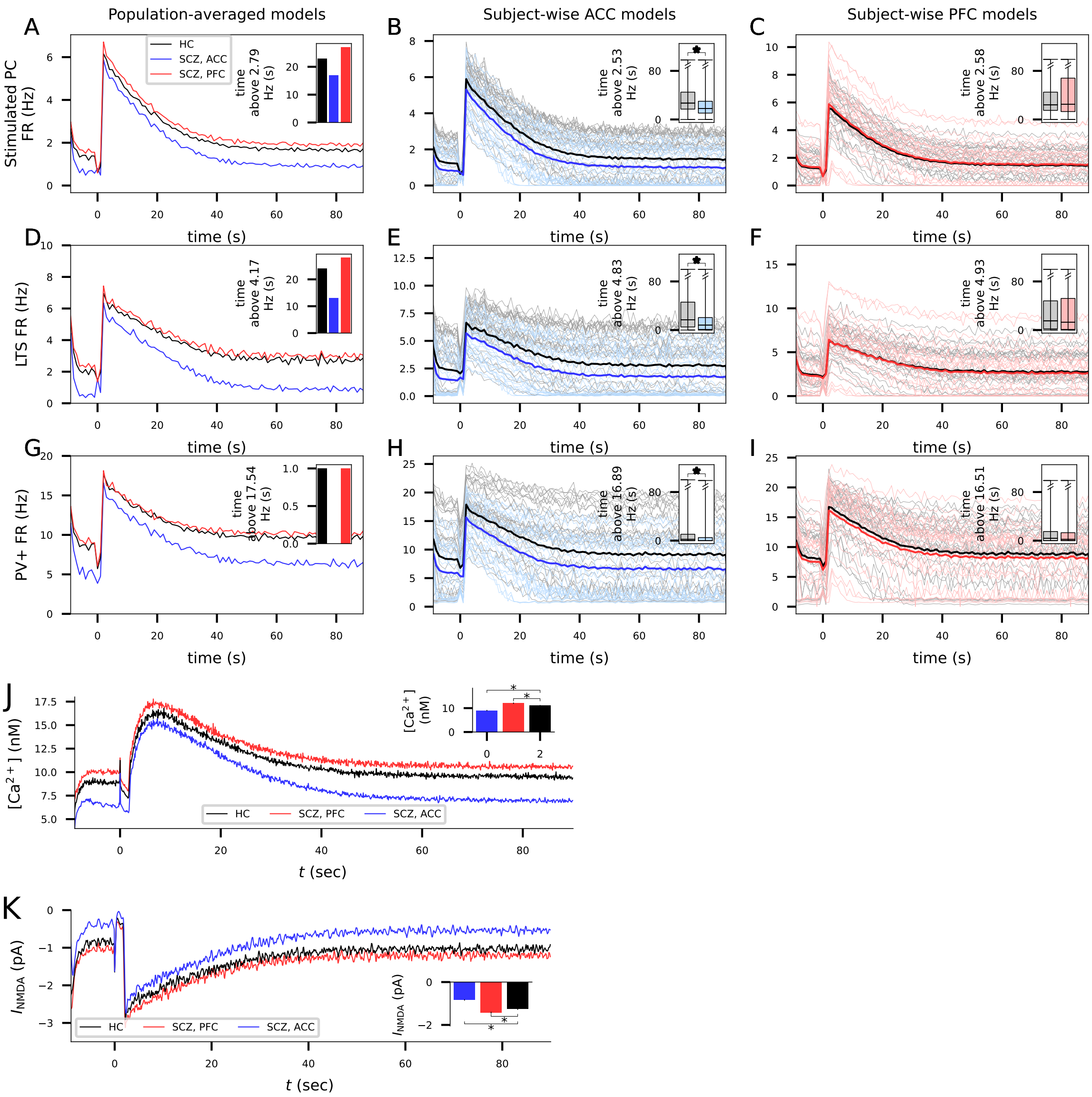

Figure S4: **ACC-like variants of ion-channel expression in pyramidal cells *and interneurons* decrease the network response to the WM-inducing stimulus**. **A**–**I**: The simulations of Fig. 3 were repeated so that the SCZ-associated parameter changes affected also the inhibitory neurons. Namely, the KCNB1 and SCN1B expression data affected the $\bar{g}_{\mathrm{K,DR}}$ and $\bar{g}_{\mathrm{Na,t}}$ parameters in all neuron populations and the CACNA1C, CACNA1D, and HCN1 expression data affected the $\bar{g}_{\mathrm{Ca,L-type}}$ and $\bar{g}_{\mathrm{Ih}}$ parameters in both pyramidal cells and LTS neurons. In the simulations of Fig. 3, in contrast, these parameter changes were only applied to the pyramidal cell population. See Fig. 3 for details. **J**–**K**: The simulations of Fig. 4A–B were repeated so that the SCZ-associated parameter changes affected also the inhibitory neurons. The Ca^{2+} concentration (J) and the NMDAR-mediated currents (K) in the pyramidal cells were smaller under ACC-like expression-level alterations and slightly larger under PFC-like expression-level alterations compared to HC. See Fig. 4A–B for details.

medRxiv preprint doi: https://doi.org/10.64898/2026.06.24.26356367; this version posted June 30, 2026. The copyright holder for this preprint (which was not certified by peer review) is the author/funder, who has granted medRxiv a license to display the preprint in perpetuity.
It is made available under a CC-BY 4.0 International license .

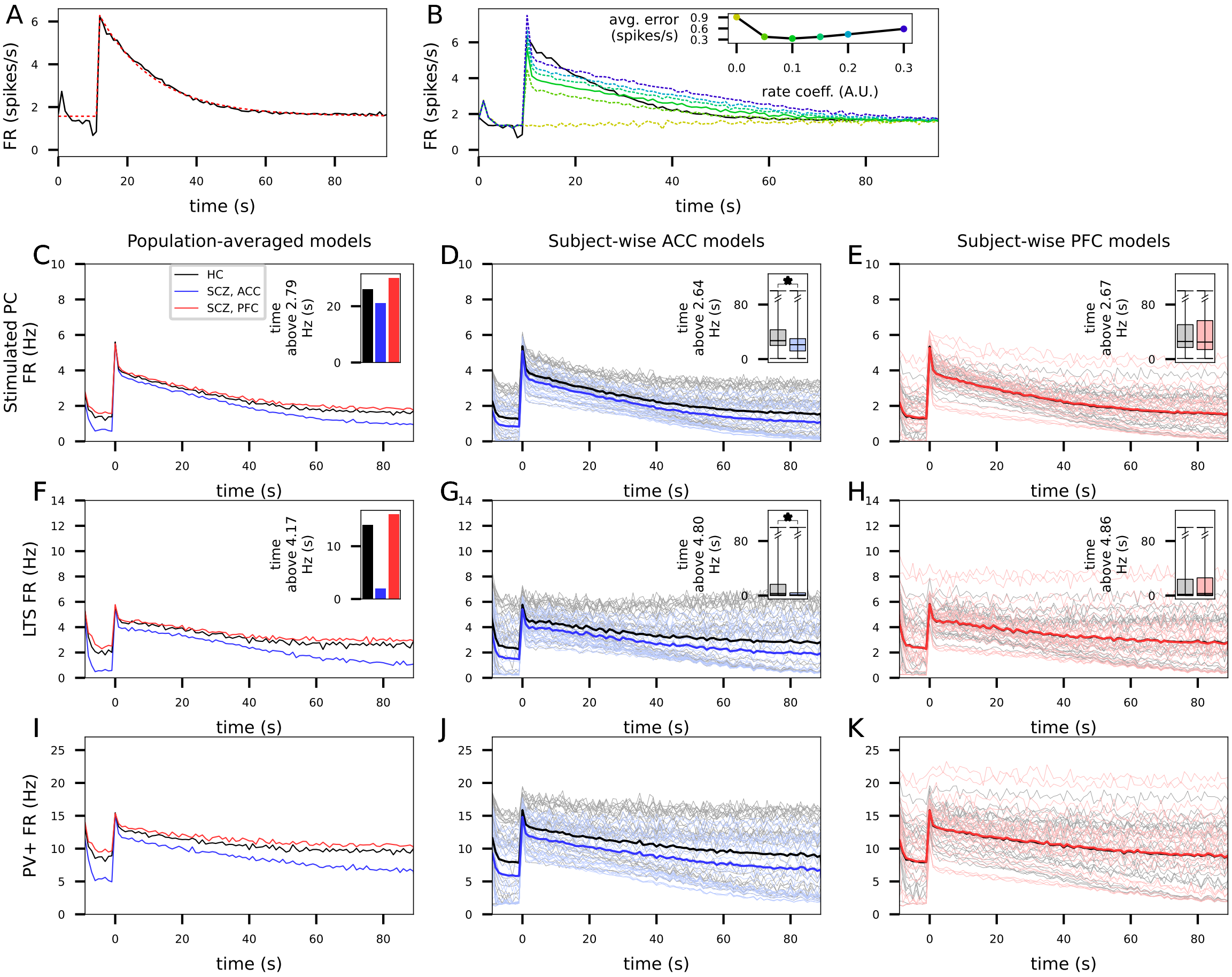

Figure S5: **ACC-like variants of ion-channel expression in pyramidal cells and interneurons decrease the network response to external stimuli that mimic the increased firing caused by the WM-inducing stimulus**. **A**: An exponential decay constant of 16 s fits the additional firing caused by the WM-induced stimulus in HCs. The black curve shows the FR of the stimulated pyramidal cells in the experiment of Fig. 3A, and the red dashed curve shows the best fit to the curve. **B**: The network with external WM-like stimulation with a decay constant of 16 s fit best to the default WM simulations when the additional input rate was 10% (at its peak) from the baseline input rate. The black curve shows the FR of the stimulated pyramidal cells in the experiment of Fig. 3A, and the coloured curves show the corresponding FR in the experiments where the HCN channel-modulating mGluR-mediated stimulus was replaced by an additional, external AMPAR-mediated synaptic stimulus that decayed with a decay constant τ=16 s and had a peak amplitude of 0% (no external stimulus; yellow dashed), 5% (green dashed), 10% (green), 15% (cyan dashed), 20% (light blue dashed), or 30% (blue dashed) of the baseline input rate. Inset: The average error in FR between default model and the model with external stimuli. The simulations with a 10% relative input rate fit best to the FR dynamics in the default network model of Fig. 3A. **C–M**: The simulations of Fig. 3 were repeated in the network where external WM-associated stimulus replaced the HCN channel-mediated persistent firing. See Fig. 3 for details.

medRxiv preprint doi: https://doi.org/10.64898/2026.06.24.26356367; this version posted June 30, 2026. The copyright holder for this preprint (which was not certified by peer review) is the author/funder, who has granted medRxiv a license to display the preprint in perpetuity.
It is made available under a CC-BY 4.0 International license .

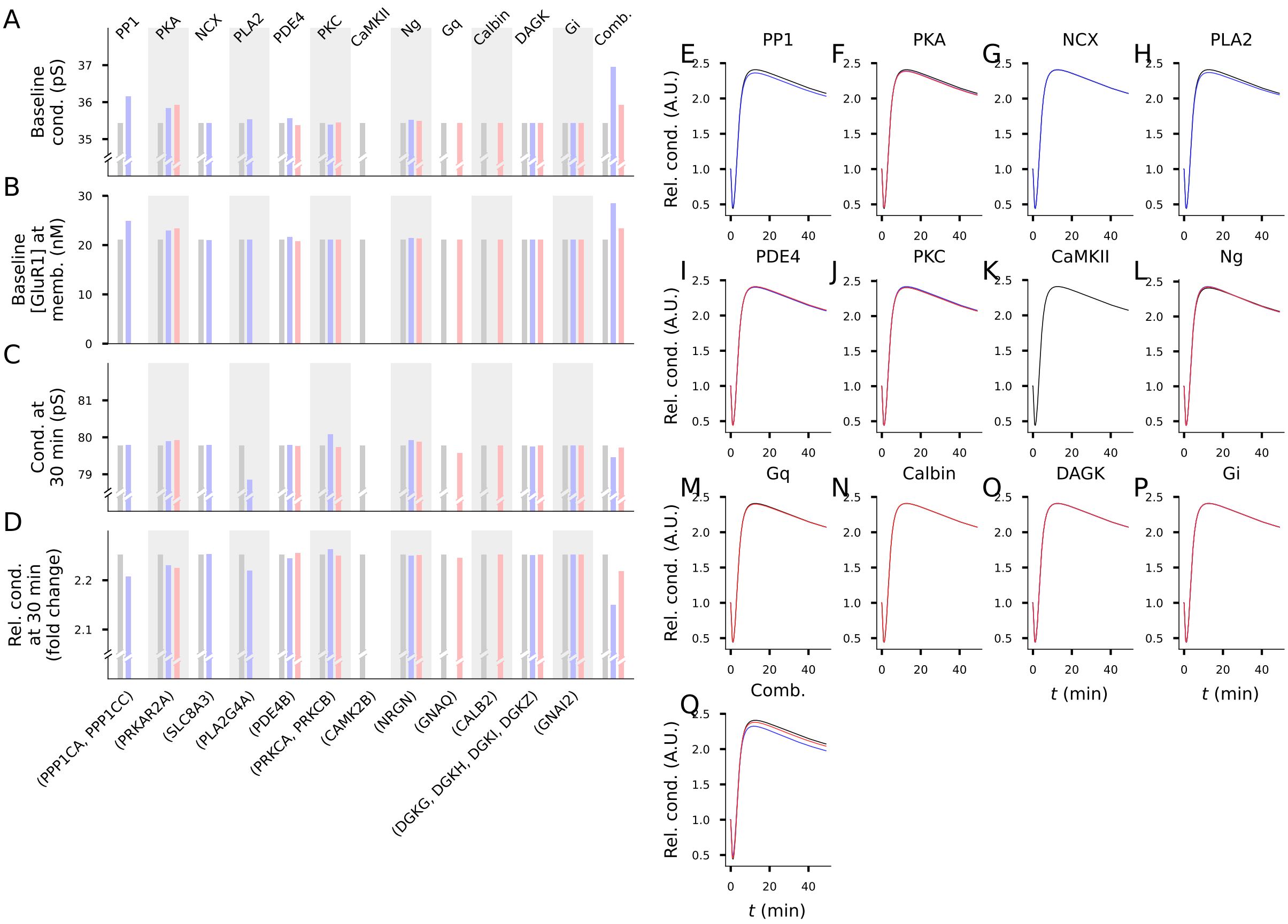

Figure S6: **Altered expression of plasticity-regulating genes in SCZ affects the baseline synaptic conductance and decreases the amplitude of the potentiation at t=30 min after stimulus onset. A**: The baseline synaptic conductance in the default model (representing HC) and in the models where expression of plasticity-regulating genes was altered according to SCZ data from ACC (blue) or PFC (red). The different columns represent different alterations of the model parameters as shown in Table 2: the concentration of PP1 was affected by expression levels of PPP1CA and PPP1CC in the ACC, PKA concentration by expression of PRKAR2A, NCX concentration by expression of SLC8A3 in the ACC, PLA2 concentration by expression of PLA2G4A, PDE4 concentration by expression of PDE4B, PKC concentration by expression of PRKCA in the ACC and PRKCB in the PFC, CaMKII concentration by expression of CAMK2B, Ng concentration by expression of NRGN, G-q concentration by expression of GNAQ, calbindin concentration by expression of CALB2 in the PFC, DAGK concentration by expression of DGKG and DGKI in the ACC, DGH in the PFC, and DGKZ in both ACC and PFC, and G-i concentration by the experession of GNAI2. The last column represents the data from the combined simulations where all expression level changes of Table 2 were implemented in the models. **B**: The baseline concentration of membrane-inserted GluR1 in the simulations of panel (A). **C**: The synaptic conductance 30 min after the onset of the 16 Hz×100 s stimulus in the simulations of (A). **D**: The relative (normalized by the baseline conductance shown in (A)) synaptic conductance 30 min after the stimulus onset. **E–Q**: The relative synaptic conductance time courses in the simulations of altered expression of plasticity-regulating genes.

medRxiv preprint doi: https://doi.org/10.64898/2026.06.24.26356367; this version posted June 30, 2026. The copyright holder for this preprint (which was not certified by peer review) is the author/funder, who has granted medRxiv a license to display the preprint in perpetuity.
It is made available under a CC-BY 4.0 International license .

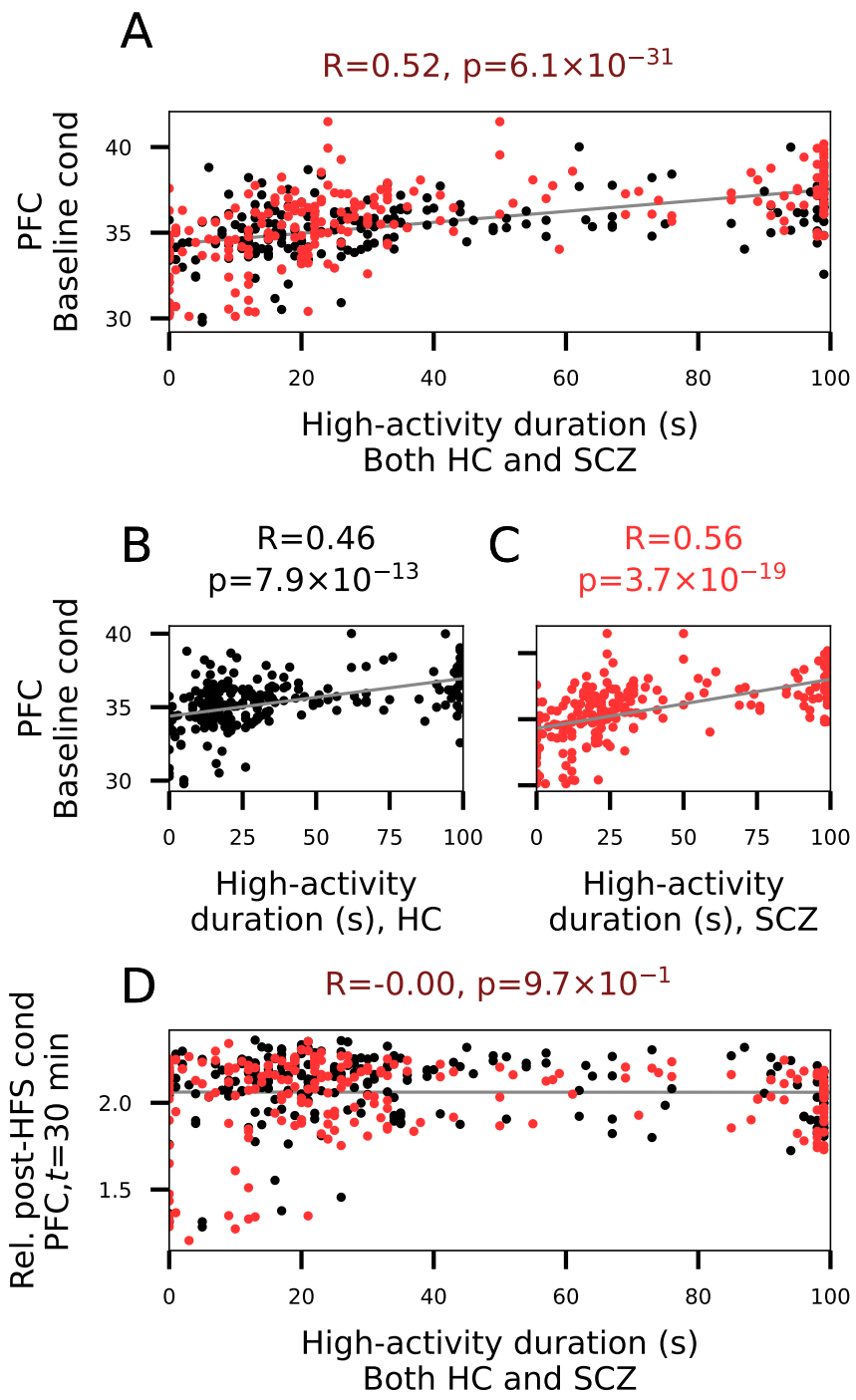

Figure S7: **Correlations between alterations in the features of the predicted plasticity and the sustained firing in the PFC. A–E**: The experiments of Fig. 5C–G repeated for the gene expression data from the PFC. See Fig. 5 for details.

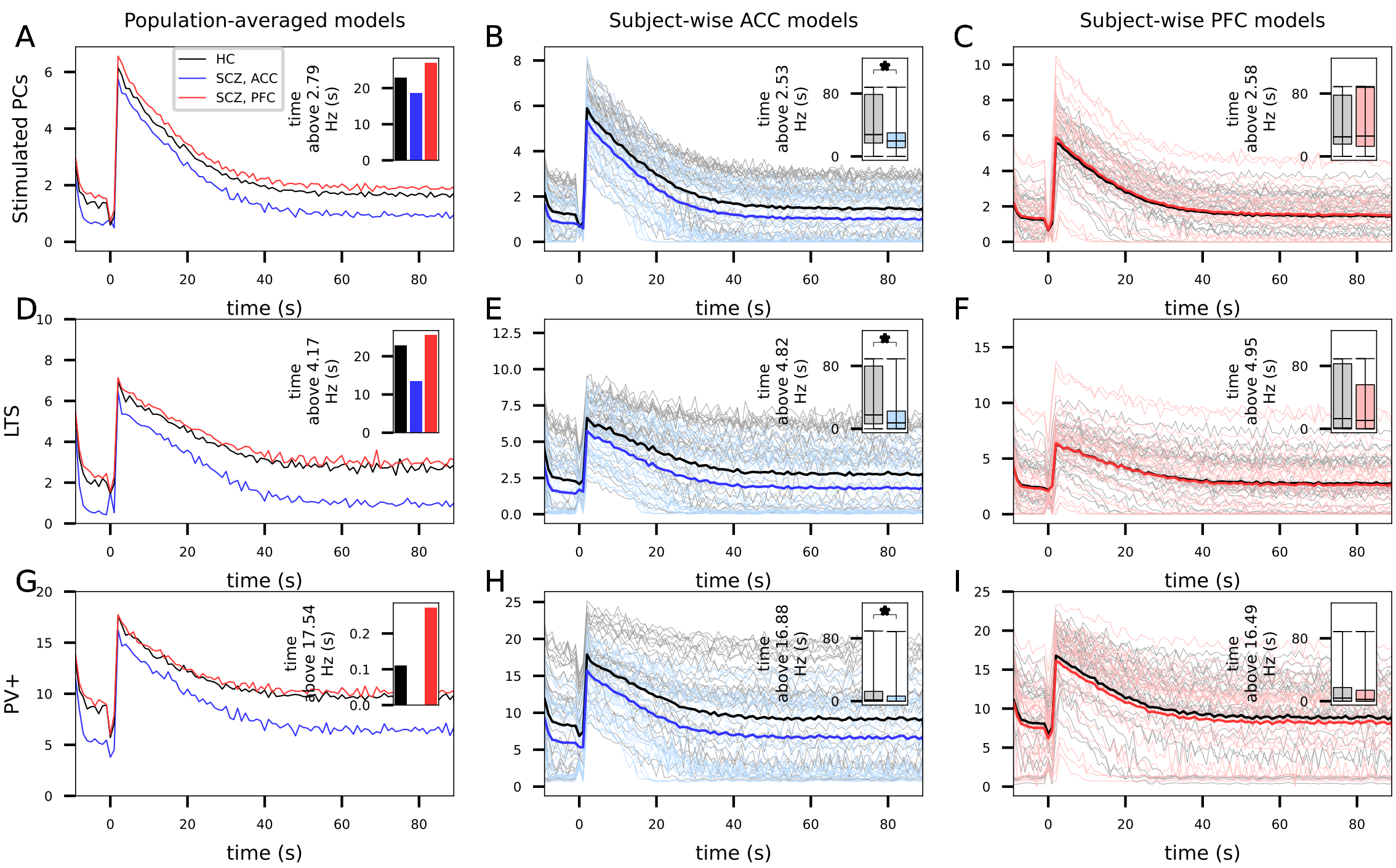

Figure S8: **ACC-like variants of ion-channel expression in pyramidal cells and interneurons, combined with alterations of baseline synaptic conductance predicted by the biochemically detailed model [Mäki-Marttunen et al., 2024], decrease the network response to the WM-inducing stimulus**. Here, all the simulations of Fig. S4 were repeated so that the baseline conductance of excitatory-to-excitatory synapses was altered according to the simulated data from Fig. 5. In the population-averaged simulations of (A), (D), and (G), the E→E synaptic conductance was 4.3% (ACC) or 1.4% (PFC) larger in SCZ than HC in addition to the parameter changes of Table 1, and in the personalized models (B–C), (E–F) and (H–I), the synaptic conductance was altered according to the subject-wise simulated data illustrated in the left insets of Fig. 5A–B. See Fig. S4 for details.

medRxiv preprint doi: https://doi.org/10.64898/2026.06.24.26356367; this version posted June 30, 2026. The copyright holder for this preprint (which was not certified by peer review) is the author/funder, who has granted medRxiv a license to display the preprint in perpetuity.
It is made available under a CC-BY 4.0 International license .

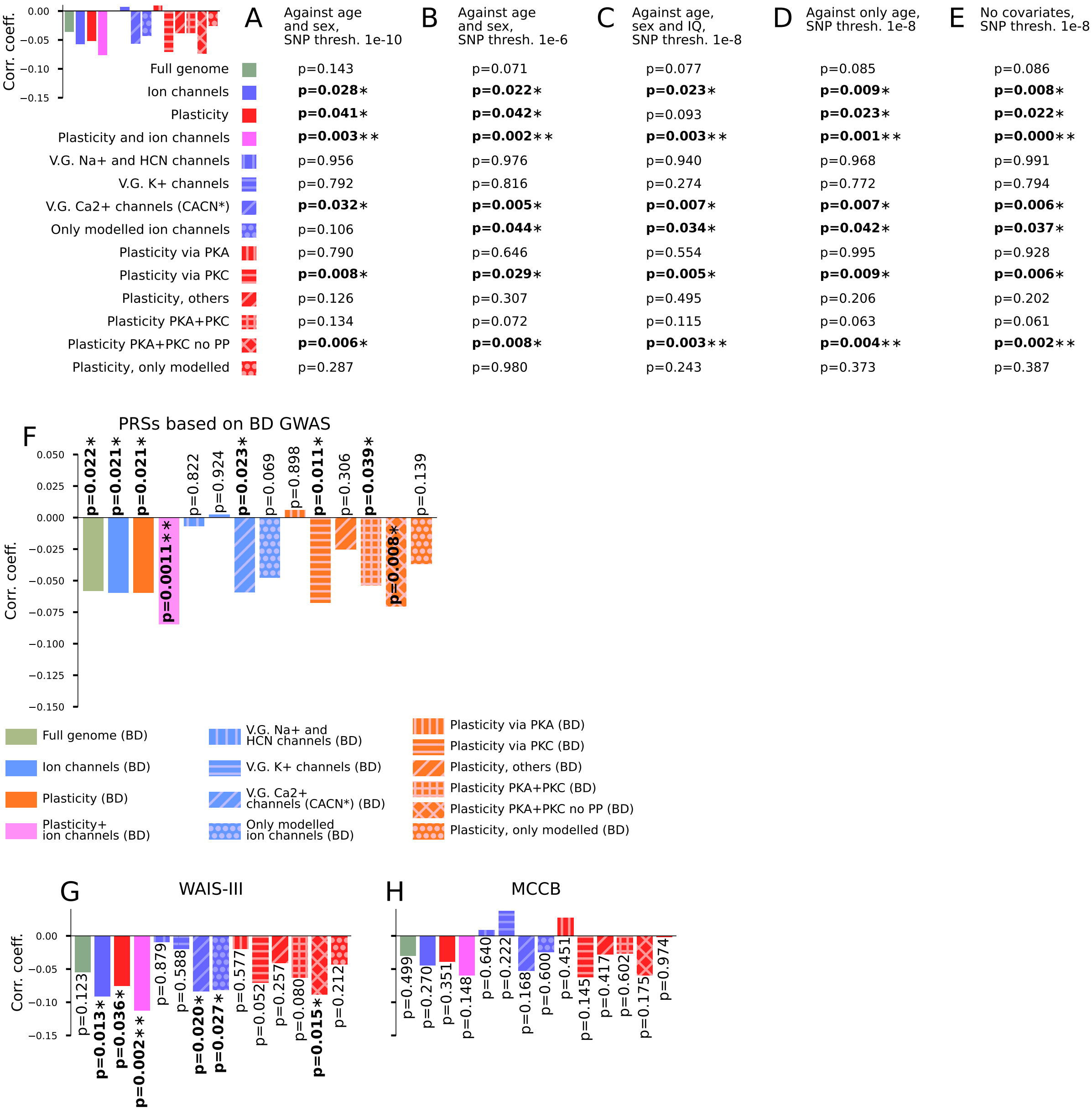

Figure S9: **Results from the genotype-phenotype analysis are robust against differences in the set of covariates but depend on the cohort.** Top left: The correlation coefficients of the PRSs and the WM performance (replotted from Fig. 6A). **A–B**: The analysis of Fig. 6A repeated using a p-value threshold of 1×10^{-10} (A) or 1×10^{-6} (B) for the SNP inclusion. See Fig. 6A for details. **C–E**: Results from the statistical tests when different sets of covariates were used. Statistically significant ($p<0.05$) associations marked with asterisks; the double asterisks indicate significance when Bonferroni-corrected for multiple tests ($p<0.05/14$). **F**: The analysis of Fig. 6A repeated using the BD-PRSs calculated from [O'Connell et al., 2025]. **G**: The analysis of Fig. 6A repeated using only the cohort where WAIS-III version of the LNS task was used. **H**: The analysis of Fig. 6A repeated using only the cohort where MCCB version of the LNS task was used.

medRxiv preprint doi: https://doi.org/10.64898/2026.06.24.26356367; this version posted June 30, 2026. The copyright holder for this preprint (which was not certified by peer review) is the author/funder, who has granted medRxiv a license to display the preprint in perpetuity.
It is made available under a CC-BY 4.0 International license .

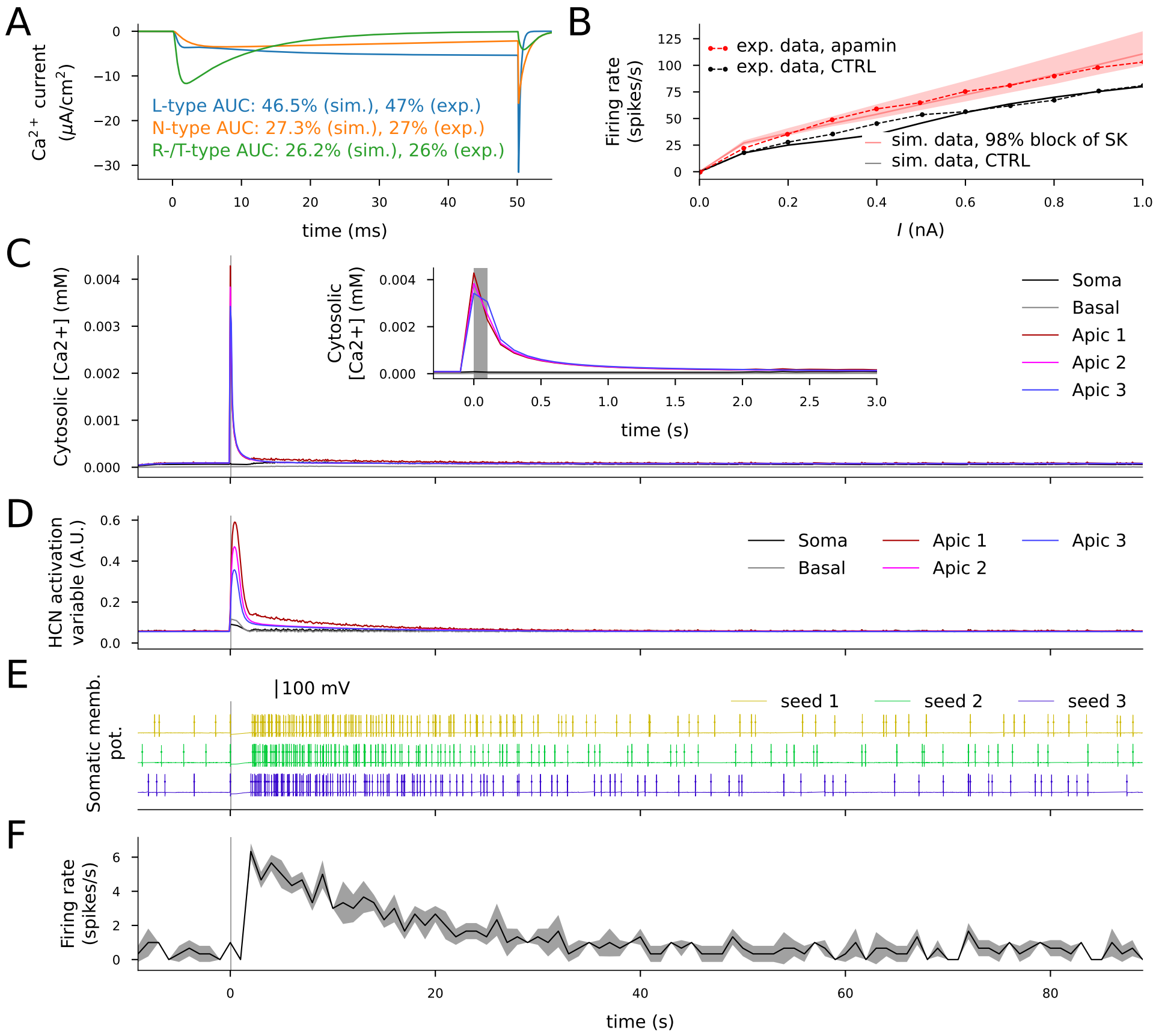

Figure S10: **The pyramidal cells without a hot zone of Ca^{2+} channels behave similarly to those with the hot zone in response to somatic current injection but display a dampened intracellular Ca^{2+} response in the apical dendrite in response to the WM-inducing stimulus. A–F**: The experiments of Fig. 8C–H repeated in pyramidal cells without the hot zone of Ca^{2+} channels. The voltage clamp response (A) and the f-I curves (B) were similar to those of the pyramidal cells with the hot zone of Ca^{2+} channels (Fig. 8C–D), but the WM-inducing stimulus caused a smaller Ca^{2+} response (C) and HCN channel activation (D) than in the pyramidal cells with the hot zone (Fig. 8E–F). This mildly increased the AP firing in response to the WM-like stimulus in the pyramidal cells without the hot zone of Ca^{2+} channels (E–F) compared to those with he hot zone (Fig. 8G–H).
